# Acute transverse myelitis in an adult-patient with underlying ulcerative colitis: a case report

**DOI:** 10.1186/s12876-022-02230-z

**Published:** 2022-04-01

**Authors:** Yi Yang, Yan Zhang

**Affiliations:** Department of Gastroenterology, West China Hospital, Sichuan University, No. 37 Guoxue Road, Chengdu, 610041 China

**Keywords:** Acute transverse myelitis, Ulcerative colitis, Mesalazine

## Abstract

**Background:**

Ulcerative colitis (UC) is an idiopathic inflammatory bowel disease that limits to colon mucosa, which characterised by relapsing and remitting abdominal pain and diarrhea. Neurological complications in UC patients are usually underestimated. The most frequently reported neurological disorders associated with UC are peripheral neuropathy, cerebrovascular disease and demyelinating disease. However, acute transverse myelitis (TM) is rarely reported in UC patients.

**Case presentation:**

We report a case of a 39-year-old man presented with fatigue, muscle weakness, numbness in the lower limbs and fingers with underlying UC. Laboratory results revealed elevated neutrophil count, high-sensitivity C-reactive protein and erythrocyte sedimentation rate. Strip-shaped high signal intensity was identified in the cervical and thoracic spinal cord on T2-weighted magnetic resonance imaging. Acute TM was diagnosed. Significant improvements after intravenous high-dose methylprednisolone were observed.

**Conclusion:**

We speculate that acute TM may be the extraintestinal manifestation of UC, which may be related to the abnormalities of cell-mediated and humoral immunity rather than the side effect of mesalazine.

## Background

Ulcerative colitis (UC) is an idiopathic inflammatory bowel disease characterised by relapsing and remitting inflammation [1]. Extra-intestinal manifestations (EIMs) of UC may include multiple organs such as joints, skin, mouth and eyes. Neurological complications in UC patients are rare. In a retrospective, observational population based-cohort including 772 patients with UC or Crohn’s disease (CD), the cumulative incidence rate of peripheral neuropathy was 0.7% after 10 years [2]. In a different retrospective computerised search, 3% of inflammatory bowel disease (IBD) patients had neurologic involvement [3]. The most often reported neurological disorders associated with UC are peripheral neuropathy, cerebrovascular disease and demyelinating disease [4]. This paper describes an unusual case of a male patient with UC who developed acute transverse myelitis (TM).

## Case presentation

A 39-year-old man presented with hematochezia and diarrhea in 2015, and UC with left-sided colitis was diagnosed in our hospital. Mesalazine was prescribed from then on. His colitis was quiescent except for several flares between 2015 and 2019 when consuming wine and spicy food. He had no relapse since 2019.

In January 2021, he was admitted to a different hospital with fatigue and muscle weakness in upper and lower limbs. After 6 days, he developed dyspepsia, and numbness in the lower limbs and fingers. Neurological examination showed normal cortical function, cranial nerve function and muscle tension of extremities, but fourth-fifth weakness of both arms and legs, pathologically brisk deep tendon reflexes of lower limbs, positive Hoffman sign, and numbness below buttocks. In the interim, he had no gastrointestinal symptoms. His personal and family history was not important for this case.

Laboratory results revealed elevated neutrophil count (6.43 × 10^9^/L), high-sensitivity C-reactive protein (26.99 mmol/L) and erythrocyte sedimentation rate (34 mm/h). Tests for hepatitis B virus, hepatitis C virus, human immunodeficiency virus and syphilis were negative. Serologic tests for antinuclear antibodies, anti-extracted nuclear antibodies and anti-dsDNA antibodies were also negative. Cerebrospinal fluid (CSF) pressure was normal but CSF contained karyocyte count of 140.0 × 10^6^/L and protein content of 1.30 g/L, in which the level of IgG increased to 192.00 mg/L. *Cryptococcus* detected by ink stain was not found in CSF and the test for DNA of *Mycobacterium tuberculosis* was negative. Metagenomic next-generation sequence for pathogen detection also had negative results. Serum antibodies against aquaporin-4, myelin oligodendrocyte glycoprotein, myelin basic protein and glial fibrillary acidic protein were negative. There was no clear lesion confirmed by computed tomogram (CT) of the brain. Chest CT revealed obsolete lesions, which ruled out pneumonia. Strip-shaped high signal intensity was identified in the cervical and thoracic spinal cord on T2-weighted magnetic resonance imaging (MRI). As the colitis remained quiescent, no pertinent examination of UC was performed.

The patient was diagnosed with acute TM and was straight away started on intravenous high-dose methylprednisolone (1000 mg daily) for 3 days. Significant improvements in fatigue and extremity muscle weakness were observed. The dosage was subsequently decreased to 500 mg/d and then to 240 mg/d after 3 days. On the day of discharge, the patient was switched to oral prednisone at 60 mg daily and followed the tapering schedule of prednisone (dosage reduction by 5 mg per week). One week later, he was able to walk with no assistance. Nevertheless, numbness in lower limbs and fingers still remained. During hospitalisation, he continued to take mesalazine as before (3 g daily).

Subsequently, he visited our outpatient clinic for advice from the gastroenterologist’s. White cell count, C-reactive protein level and erythrocyte sedimentation rate were within normal limits. Colonoscopy showed mild proctitis. The possibility that the acute TM might be the side effect of mesalazine was considered, so oral-mesalazine was discontinued, and mesalazine suppositorics were added. In April 2021, oral-mesalazine was added again, due to no relapse of TM. After prednisone withdrawal on May 24, 2021, he only had mild numbness occasionally in the right leg. Throughout the 2 months of follow-up when he took only mesalazine (2 g daily), abdominal pain, hematochezia and diarrhea were not reported.

## Discussion and conclusions

Patients with UC can present with neurological manifestations involving the central nervous system and peripheral nervous system, which include demyelinating and axonal neuropathies, sensorineural hearing loss, thromboembolism and cerebrovascular disease, epilepsy, myelopathy and cerebral vasculitis [5]. The pathophysiological association of neurological disorders with UC has not been established. It could involve diverse mechanisms: (1) nutritional deficiencies (vitamin B_12,_ vitamin E, folic acid and nicotinamide), (2) toxic metabolic agents, (3) infections as a complication of immunosuppression, (4) side effects of medication or surgery, (5) thromboembolism, (6) immunological abnormalities [6].Nonetheless, the myelitis occurring in underlying UC has seldom been reported in published literature [3, 7, 8]. Ray et al. reported that a 32-year-old woman who had long-standing and quiescent UC presented with paresthesia, sensory loss and muscle weakness. MRI confirmed TM in the cervical spinal cord. However, it was thought that TM and UC might co-exist by chance as both of them are immunologically mediated [7]. Gibb et al. [8] reported a 40-year-old man developed paresthesia and spastic paraparesis after an earlier phase of active UC and colectomy, and the neurological lesion was regarded as a non-specific immune response. Referring to another case, a 50-year-old woman with UC had progressive myelopathy manifested by paraparesis, increased tendon reflexes and bilateral extensor plantar response while the relationship between TM and UC was not explained [3]. Our report is the fourth one that endeavors to demonstrate the association of TM with UC.

Acute TM is a rare neurological disorder of the spinal cord that presents with motor, sensory and autonomic dysfunction. It remains etiologically unknown. A number of case reports have described TM after vaccination although the cause has not been established. In some researches, infectious agents are also considered to be triggering factors of TM. Systemic inflammatory autoimmune diseases, such as systemic lupus erythematosus (SLE), Sjogren’s syndrome and sarcoidosis, associated with TM have been reported [9–11]. In our case, the association of TM with underlying UC remains unclear. No evidence of infection was found. The side effect of mesalazine was also suspected. According to the prescribing information of mesalazine, TM has been reported voluntarily. But so far, no literature has been reported that TM was secondary to mesalazine. A paper that reported a patient diagnosed with TM after sulfasalazine treatment had been published [12]. In our case, the patient continued to take mesalazine throughout the TM therapy. After prednisone withdrawal and mesalazine being recontinued for 2 months, the patient was clinically stable with no TM relapse, which suggested that the acute TM was not a side effect of mesalazine.

The extraintestinal manifestations of IBD are believed to stem from an extension of antigen-specific immune response from the intestine or to be independent inflammatory events by the presence of IBD. There is also an overlap in genetic risk loci for EIMs and IBD [13]. In SLE-related TM, tissue sampling of the spinal cord exhibits vasculitic lesions. These observations verify that SLE-related TM is an abnormality of cell-mediated and humoral immunity [14]. Therefore, we hypothesize that acute TM is more apt to be the extraintestinal manifestation of UC. The role of autoantibodies in patients with neuromyelitis optica and recurrent TM was described and high levels of circulating antibodies may have a causative role in TM [15]. Consequently, we hypothesize that antibodies or immune complexes might play a role in UC-related TM, but the hypothesis still has no obvious evidence.

Up till the present time, there is no optimal therapy of TM in UC. According to our report, high-dose intravenous glucocorticoid treatment attained significant recovery. Intravenous steroid is typically instituted in patients with acute TM. Plasma exchange has been demonstrated to be effective in patients with severe TM who don’t improve with intravenous steroid [14]. In SLE-related TM, immunosuppressive therapy is also recommended [16].

In conclusion, this case highlights that the acute TM is presumptively associated with UC and a multidisciplinary team is essential to attain effective management. We speculate that TM is the extraintestinal manifestation of UC related to abnormalities of cell-mediated and humoral immunity, but the side effect of mesalazine ought to also be considered. With an inadequate histological basis of spinal cord and serological evidence, the association of TM with underlying UC remains in our case, ambiguous. Consequently, additional related case reports, cohort studies and research on pathogenesis are required.

## Data Availability

All data generated during this study are included in this published article.

## Abbreviations

UC: Ulcerative colitis
CD: Crohn’s disease
TM: Transverse myelitis
IBD: Inflammatory bowel disease
CSF: Cerebrospinal fluid
CT: Computed tomogram
MRI: Magnetic resonance imaging
SLE: Systemic lupus erythematosus
EIM: Extraintestinal manifestation
